# Author Correction: A novel interface for cortical columnar neuromodulation with multipoint infrared neural stimulation

**DOI:** 10.1038/s41467-024-54090-8

**Published:** 2024-11-11

**Authors:** Feiyan Tian, Ying Zhang, Kenneth E. Schriver, Jia Ming Hu, Anna Wang Roe

**Affiliations:** 1 Department of Neurosurgery of the Second Affiliated Hospital, Interdisciplinary Institute of Neuroscience and Technology, School of Medicine, Zhejiang University, Hangzhou, 310029 China; 2 Key Laboratory for Biomedical Engineering of Ministry of Education, College of Biomedical Engineering and Instrument Science, Zhejiang University, Hangzhou, 310027 China; 3 MOE Frontier Science Center for Brain Science and Brain-machine Integration, School of Brain Science and Brain Medicine, Zhejiang University, Hangzhou, 310012 China; 4 National Key Laboratory of Brain and Computer Intelligence, Zhejiang University, Hangzhou, 310058 China

**Keywords:** Striate cortex, Biomedical engineering, Lasers, LEDs and light sources

Correction to: *Nature Communications* 10.1038/s41467-024-50375-0, published online 02 August 2024

In this article the affiliation details for Feiyan Tian, Ying Zhang, Kenneth E. Schriver, Jia Ming Hu & Anna Wang Roe were incorrectly given as ‘Department of Neurosurgery of the Second Affiliated Hospital, Interdisciplinary Institute of Neuroscience and Technology, Zhejiang University, Hangzhou, 310029, China’ but should have been ‘Department of Neurosurgery of the Second Affiliated Hospital, Interdisciplinary Institute of Neuroscience and Technology, School of Medicine, Zhejiang University, Hangzhou, 310029, China’. The original article has been corrected.

