# Supplementary material for: A novel interface for cortical columnar neuromodulation with multipoint infrared neural stimulation
Source: Nat Commun. 2024 Aug 2;15:6528. doi: 10.1038/s41467-024-50375-0 (PMC11297274; doi:10.1038/s41467-024-50375-0)
Supplement: Supplementary file 1 — Supplementary Information [file 41467_2024_50375_MOESM1_ESM.pdf]

Supplementary Materials

A novel interface for cortical columnar neuromodulation with multi-point infrared neural stimulation

Supplementary method

Temperature Rise induced by INS Stimulation

There have been multiple studies of temperature rise and damage thresholds of pulsed infrared neural stimulation (INS). Findings indicate that, for stimulation paradigms similar to that described here, thresholds, estimated from histological studies fall between 0.6J/cm^2 – 1J/cm^2 (rat cortex 0.4J/cm^2 and nonhuman primate cortex[1] ~0.6J/cm^2, human cortex[2] 0.6J/cm^2, human spinal roots[3] 1.09J/cm^2, cochlea 25uJ/pulse[4]).

Studies have also examined the temperature rise due to INS. Thompson et al.[5] modelled temperature rise in rat peripheral nerve and found that 250Hz stimulation with comparable parameters (200um fiber, pulse width 100usec, 1850nm, 25uJ) produced a temperature rise of 2.3°C. We have also addressed this issue using MRI thermometry (which measures the shift in proton resonance frequency caused by temperature increase). Using the same parameters used in this study for INS stimulation in ex vivo rat brains, MRI thermometry (spatial resolution: 1 mm isotropic voxels) reveals that the spatial extent of temperature increase is quite confined and the highest ΔT (measured at the strongest voxel in response to the highest intensity of 1J/cm2, red line) plateaus below 2°C (for optogenetic stimulation[6] see Luo et al. 2023).

To ensure the temperature rise of laser will not damage tissues, there is a In the thermometry study, we converted the direct proton resonance frequency phase shift data in acquired images to temperature change according to[6,7]:

$$\Delta T = \frac{\phi(T) - \phi(T_0)}{\gamma \alpha B_0 TE} \quad (1)$$

where $\phi(T)$ is the phase map at current time point, $\phi(T_0)$ is the phase map of the baseline image which is measured at room temperature (before INS), γ here represents the gyromagnetic ratio

of hydrogen (2.67×10^8 rads per Tesla, constant), α is the PRF shift coefficient of water (-0.01 ppm per °C, constant), B_0 is the magnetic field strength (7 Tesla here), and TE is the echo time of imaging sequence (1.65 msec here). And in our condition, 1 °C $\cong$ 0.031 rads.

The temperature of the voxel with greatest phase shift at the location of the optic fiber tip was selected, and the temperature increase in the 9 isolated measurements was averaged for further analysis. ANOVA and paired-sample t-test were conducted to statistically analyze the temperature changes before and after INS, as well as the differences across different power intensities at the same time point. The data was analyzed using MATLAB.

In sum, assessments of heat induced damage via histological methods, thermometry, and modelling, all indicate that INS delivered with these parameters are non-damaging. Note also that, based on these studies, we have conducted INS in human cortex[2] and there are currently clinical trials using INS (cochlea: Richter NCT05110183, peripheral nerves: Jansen NCT04601337).

Supplementary Figures

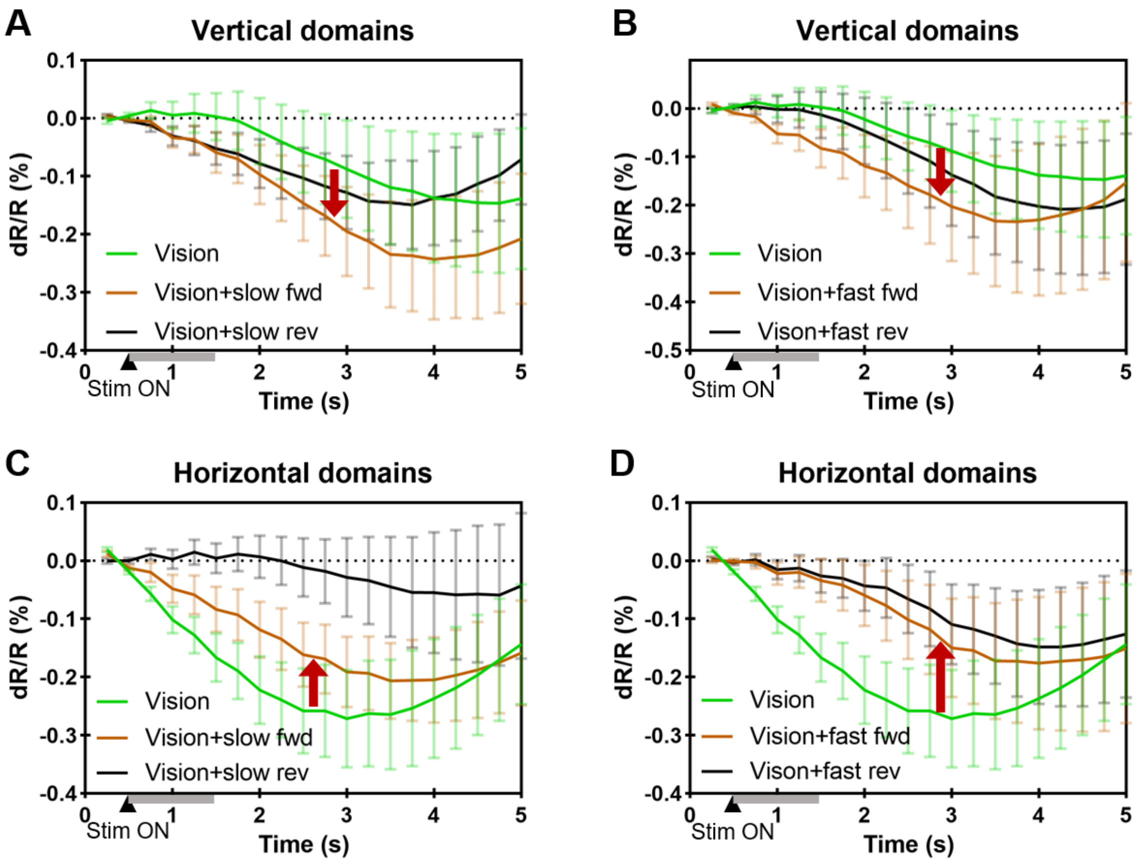

Supplementary Fig.1. Directionality. OI Response for ROIs in right panel of Fig. 5A on right hemisphere during different directions of ‘vertical INS’ on left hemisphere. (A & B) 2 directions with slow or fast speed, both enhance the vertical visual response (INS and visual stimulation are matched). (C & D) 2 directions with slow or fast speed, both reduce the horizontal visual response (INS and visual stimulation are non-matched). FWD: posterior to anterior, away from HM. REV: anterior to posterior, towards HM. All symbols are same as Figure 5. Gray bar indicates the duration of INS. N = 15 trials in (A-D), data are presented as mean values +/- SEM. INS: 1870 nm, 200 Hz, 0.3 J·cm^{-2}, pulse width 250 μs, pulse train 1 s.

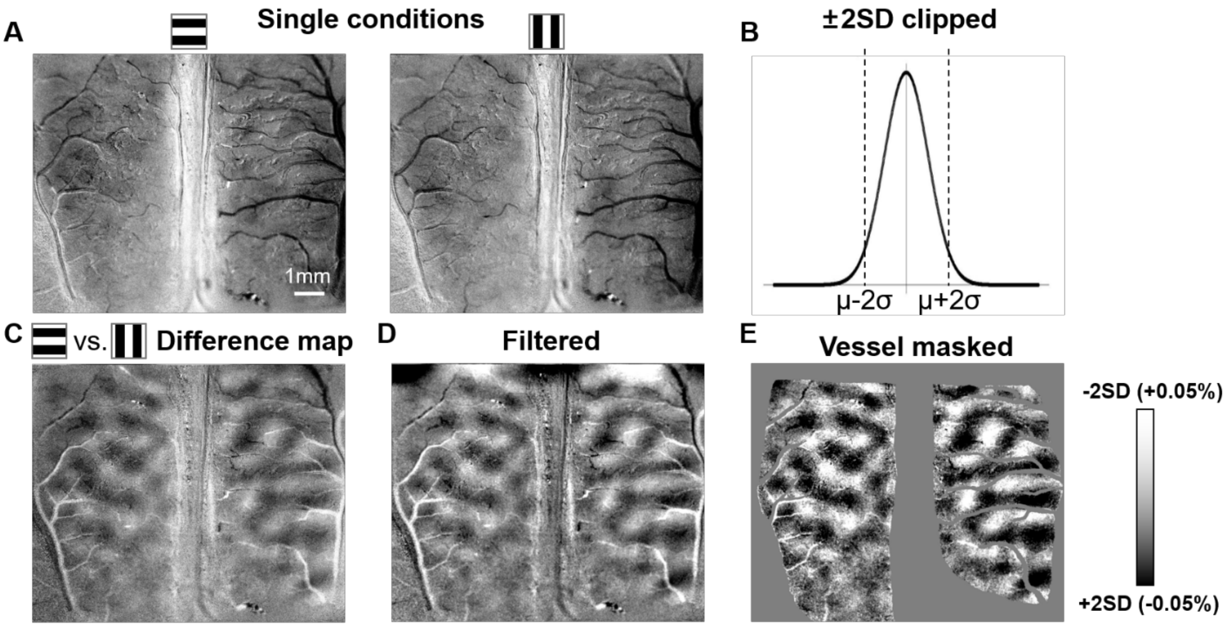

Supplementary Fig.2. Steps in processing of OI data. (A) Single condition maps or horizontal (left) and vertical (right) gratings. (B) In the grayscale distribution of an image, we ‘clip’ the distribution to remove large artifactual (e.g. dR/R > 2%) reflectances which are often due to large vascular pulsations or locations of specularity. A clip of median ± 2SD is typical. (C) Difference map of two single-condition maps. Dark: horizontal preferring. Light: vertical preferring. (D) Filtered map based on difference map, with both low and high pass (see text). (E) Following removal of large vessel pixels, the filtered map is clipped.

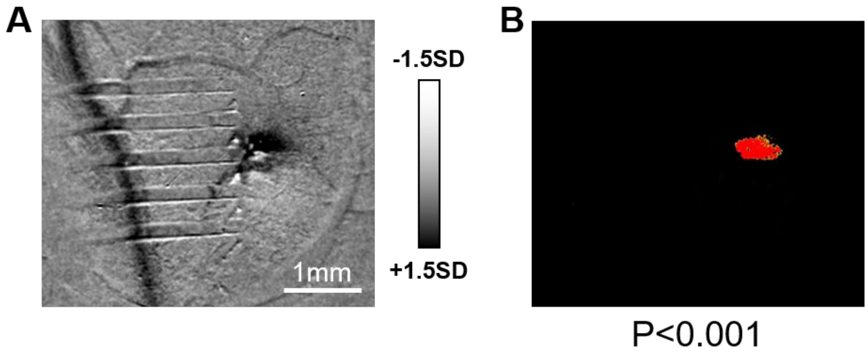

Supplementary Fig.3. The threshold used to choose activation region. (A) Original figure clipped with ±1.5SD (B) T-test map when $p<0.001$, which is the threshold we chose to indicate the activation region. The area is determined from the number of significant pixels and the diameter is the average of the long and short axes.

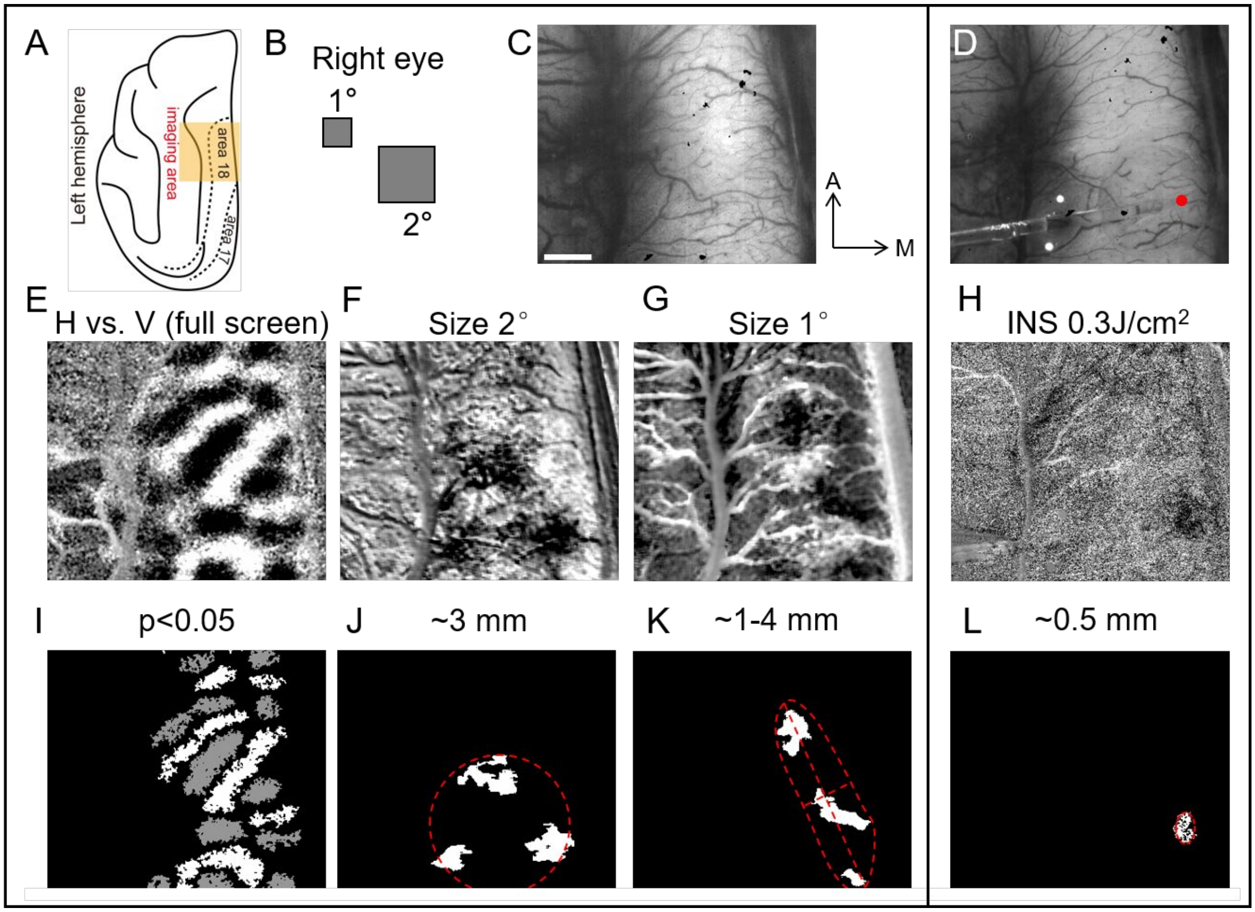

Supplementary Fig.4. Comparison of response size evoked by spot visual stimuli and single fiber INS stimulation. (A) Schematic picture of a cat cerebral cortex (based on Tanaka et al.[8]). Orange rectangle: approximate imaging field of view. Dotted lines: approximate areal borders. (B) Schematic of spot positions. 2° spot slightly more lateral than 1° spot. (C, D) Blood vessel maps recorded in the visual stimulation and INS conditions. (E, I) orientation map of 'H'-'V' full screen grating visual stimulation (binocular stimuli). (F, G) response map of 'vision'-'blank' from 2° (F), 1° (G) visual spot stimulation (monocular stimuli). (H) activation map in response to single fiber INS stimulation ('INS'-'blank', wavelength: 1870nm, frequency: 200Hz, radiant exposure: 0.3 $J \cdot cm^{-2}$, pulse width: 250us, pulse train: 0.5s). (J-L) significantly ($p<0.05$) activated pixels of F-H. Numbers at top of each panel: activation size of the cortex in the corresponding condition. Scale bar: 1mm. A: anterior, M: median.

References

1. Chernov, M. M., Chen, G. & Roe, A. W. Histological Assessment of Thermal Damage in the Brain Following Infrared Neural Stimulation. *Brain Stimulat.* **7**, 476–482 (2014).
2. Pan, L. *et al.* Infrared neural stimulation in human cerebral cortex. *Brain Stimulat.* **16**, 418–430 (2023).
3. Cayce, J. M. *et al.* Infrared neural stimulation of human spinal nerve roots *in vivo*. *Neurophotonics* **2**, 015007 (2015).
4. Goyal, V., Rajguru, S., Matic, A. I., Stock, S. R. & Richter, C.-P. Acute Damage Threshold for Infrared Neural Stimulation of the Cochlea: Functional and Histological Evaluation. *Anat. Rec. Adv. Integr. Anat. Evol. Biol.* **295**, 1987–1999 (2012).
5. Thompson, A. C., Wade, S. A., Cadusch, P. J., Brown, W. G. A. & Stoddart, P. R. Modeling of the temporal effects of heating during infrared neural stimulation. *J. Biomed. Opt.* **18**, 035004 (2013).
6. Luo, H. *et al.* Detection of laser-associated heating in the brain during simultaneous FMRI and optogenetic stimulation. *Magn. Reson. Med.* **89**, 729–737 (2023).
7. Rieke, V. & Butts Pauly, K. MR thermometry. *J. Magn. Reson. Imaging* **27**, 376–390 (2008).
8. Tanaka, S. Development and Reorganization of Orientation Representation in the Cat Visual Cortex: Experience-Dependent Synaptic Rewiring in Early Life. *Front. Neuroinformatics* **14**, (2020).
